# Impact of Topically Administered Steroids, Antibiotics, and Sodium Hyaluronate on Bleb-Related Infection Onset: The Japan Glaucoma Society Survey of Bleb-Related Infection Report 4

**DOI:** 10.1155/2017/7062565

**Published:** 2017-09-12

**Authors:** Hideto Sagara, Tetsuya Yamamoto, Kimihiro Imaizumi, Tetsuju Sekiryu

**Affiliations:** ^1^The Marui Eye Clinic, Minamisoma, Fukushima, Japan; ^2^Department of Ophthalmology, Fukushima Medical University School of Medicine, Fukushima, Japan; ^3^Department of Ophthalmology, Gifu University Graduate School of Medicine, Gifu, Japan

## Abstract

**Purpose:**

To investigate the impact of topically administered ophthalmic medications on the onset and severity of bleb-related infections.

**Methods:**

Data obtained from 104 eyes of 104 patients with bleb-related infections were analyzed. We assigned an infection stage to each eye (stage 1–4) and analyzed the onset severity.

**Results:**

Steroids and antibiotics were routinely administered to 13 (12.5%) and 42 (40.4%) eyes, respectively. The median stage of steroid-administered eyes was 3 versus 1 for eyes without steroid administration (*P* = 0.012). The median duration from surgery to infection for the steroid-administered eyes was 2.0 years versus 5.8 years for eyes without steroid administration (*P* = 0.030). The median duration from surgery to infection for the antibiotic-administered eyes was 6.4 years versus 3.9 years for eyes without antibiotic administration (*P* = 0.025). Multiple logistic regression analysis revealed that infections were severe in the steroid-administered eyes (odds ratio: 4.57). No infections developed within 16 weeks postoperatively. No relationship was detected between sodium hyaluronate and the analyzed factors.

**Conclusions:**

Topical steroid administration beyond the immediate postoperative period may affect severe and earlier onset bleb-related infections. Conversely, topical antibiotic administration may be effective in suppressing earlier onset bleb-related infections.

## 1. Introduction

Filtering surgery is the most well-known surgical procedure for glaucoma [1, 2], and antifibrotic agents, such as mitomycin C, 5-fluorouracil, and steroids, improve postoperative intraocular pressure control [3–9]. However, blebs often become thin-walled and vulnerable over time. Thereafter, complications, such as hypotony, bleb leakage, and bleb-related infections, can occur [10–13]. These complications, especially bleb-related infections, must be diagnosed early and treated as soon as possible before the condition becomes severe. If the infection is localized to the bleb, the prognosis is relatively good [14, 15]; however, if the infection extends into the vitreous and becomes panophthalmitis, it often results in blindness [10, 14, 16, 17].

Nevertheless, topical administration of a steroid may improve postoperative intraocular pressure control [6–8], but it also suppresses immunity [18, 19]; therefore, it may exacerbate infection. Besides, steroid treatment is unfortunately necessary in patients who have an ocular disease. Prophylactic antibiotic treatment may be effective in preventing infection, although some reports suggest that continuous postoperative antibiotic use paradoxically increases bleb-related infection risk [20, 21]. Therefore, even among glaucoma specialists, opinions differ regarding postoperative antimicrobial prophylaxis [22]. Moreover, it has been reported that sodium hyaluronate eye drop administration protects vulnerable blebs and prevents late-onset bleb leaks [23]. Therefore, topical sodium hyaluronate administration may also be effective in preventing bleb-related infections.

The long-term effects of steroids, antibiotics, and sodium hyaluronate on bleb-related infections are not well known. In this study, as a part of the Japan Glaucoma Society Survey of Bleb-related Infection (JGSSBI) [14, 24, 25], we investigated the impact of steroids, antibiotics, and sodium hyaluronate on bleb-related infections.

## 2. Materials and Methods

### 2.1. Study Design and Patient Eligibility

Patients and JGSSBI have been previously described in detail [14, 24]. Briefly, 82 clinical centers participated in this prospective study, including 21 university hospitals, 23 public hospitals, and 38 private ophthalmology clinics. The observation period was five years, ending on March 31, 2010. Institutional review board approval was obtained at each institution except for 36 clinics, each of which received approvals for the study protocol from the Ethical Review Board of Gifu University Hospital.

Initial bleb-related infections in 104 eyes of 104 patients (76 men and 28 women) during the study period were identified. The inclusion criteria were as follows: (1) infections developed not earlier than 4 weeks postoperatively, and (2) the duration from the most recent visit to infection being detected was not greater than 6 months. We investigated the impact of eye drops and eye ointments containing steroids, antibiotics, or sodium hyaluronate that were administered prior to the bleb-related infection onset. The investigated factors were onset severity, the time from last glaucoma surgery to infection onset, intraocular pressure (IOP), visual acuity (VA), and detected bacteria. Eyes were excluded if they had been administered with topical steroids, antibiotics, or sodium hyaluronate in short-term for acute eye diseases. Eyes that were enucleated, eviscerated, or had developed phthisis bulbi were excluded from IOP analysis.

Each infection was classified into one of three stages [26]: stage I denoted infections confined to the bleb site with a mild cell reaction in the anterior chamber; stage II denoted infections where the anterior chamber was the main locus and the vitreous was not involved; and stage III denoted infections involving the vitreous. Stage III was subdivided into stages IIIa and IIIb [27]: stage IIIa denoted mild involvement of the vitreous and stage IIIb denoted more advanced involvement. The staging into subcategory IIIa or IIIb was performed based on indirect ophthalmoscopy of the fundus and the presence of vitreous opacities on B-mode echography.

We reassigned infection stages I, II, IIIa, and IIIb as stages 1, 2, 3, and 4, respectively, and analyzed the relationship between infection severity stage and the other factors listed above. Other analyzed factors were bleb morphology, bleb vascularity, and history of bleb leakage prior to infection. The bleb morphology was classified based on the following characteristics [27–29]. Cystic blebs had a thin and polycystic appearance. Diffuse blebs had good filtration and were diffused. Encapsulated blebs had a localized, fluid-filled cavity of hypertrophied Tenon's capsules. Flat blebs had poor filtration with flat and engorged surface blood vessels. Bleb vascularity was classified with the absence area of bleb surface vessels; vascular (0%), partial avascular (<50%), and avascular (≥50%), respectively. For calculating visual acuity, visual acuity <logMAR 0.01 was treated as follows: counting fingers was recorded as 0.004, hand motion as 0.002, light perception as 0.001, and no light perception as 0.0004 in logMAR.

### 2.2. Statistical Analysis

Wilcoxon signed-rank tests were used to compare IOP and logMAR VA before and after infection onset. Mann–Whitney *U* tests were used to compare categorical variables between two groups. Kruskal–Wallis tests were used to compare categorical variables between three groups, and the Steel test was used for multiple comparisons. Multiple logistic regression analysis was performed with a backward, stepwise approach to identify factors associated with infection severity and the duration from surgery to infection onset. Statistical significance was set at probability (*P*) values <0.05. All statistical analyses were performed using the statistical software EZR (Easy R, version 1.32) [30].

## 3. Results

### 3.1. Epidemiology: Prevalence and Presenting Characteristics

The mean ± standard deviation (SD) patient age at the time of infection onset was 58.0 ± 17.7 years. The mean ± SD interval between last glaucoma surgery and infection onset was 6.4 ± 5.7 years (range, 0.3–41.4 years). There were no eyes with bleb-related infections in the immediate postoperative period (within 16 weeks postoperatively). Steroids were topically administered to 13 eyes (12.5%): 0.1% betamethasone eye drops in 6 eyes (5.8%), 0.02% or 0.1% fluorometholone eye drops in 6 eyes (5.8%), and a combination ointment of 0.35% fradiomycin sulfate and 0.1% methylprednisolone in 1 eye (1.0%; Table 1). The glaucoma subtypes of the 13 eyes which were administered topical steroids were secondary glaucoma in 7 eyes, primary open-angle glaucoma in 3 eyes, developmental glaucoma in 1 eye, and unknown glaucoma subtype in 2 eyes. The 7 secondary glaucoma eyes included steroid-induced glaucoma in 1 eye (primary disease unknown), uveitic glaucoma associated with sarcoidosis in 1 eye, postkeratoplasty glaucoma in 1 eye, glaucoma secondary to essential iris atrophy in 1 eye, glaucoma secondary to Posner–Schlossman syndrome in 1 eye, and unknown subtype in 2 eyes. At the most recent visit before infection onset, topical steroids were administered to improve postoperative intraocular pressure control in 5 eyes, to suppress inflammation in 3 eyes, to suppress an immune response in 1 eye after keratoplasty, and for an undetermined purpose in 4 eyes. No relationship was detected between the therapeutic purposes and the analyzed factors. Of the 13 eyes for which topical steroids were administered, antibiotics were administered simultaneously for 10 (76.9%) eyes, the duration from the most recent visit to infection being detected was longer than two weeks for 11 (84.6%) eyes, and the duration was longer than one month for 5 (45.5%) eyes.

Antibiotics were topically administered to 42 eyes (40.4%). Overall, 40 eyes (38.5%) were treated with new-generation quinolones; 0.5% levofloxacin eye drops alone were administered to 28 eyes (26.9%; Table 1). Other antibiotic eye drops were administered to 8 eyes (7.7%; Table 2). 0.3% ofloxacin ointment only or in combination with other antibiotic eye drops was administered to 6 eyes (5.8%).

### 3.2. Impact of Topically Administered Steroids, Antibiotics, and Sodium Hyaluronate on Infection Severity and Duration from the Surgery to Infection Onset

**Figure 1:**
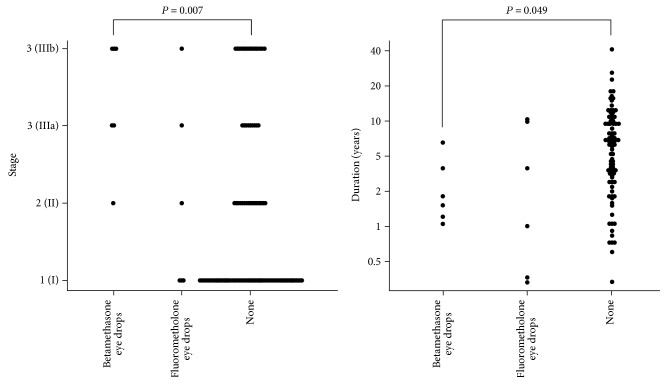
Multiple comparisons of topical steroid administration effects on infection onset severity and duration from the last glaucoma surgery to the bleb-related infection onset (Steel test).

**Figure 2:**
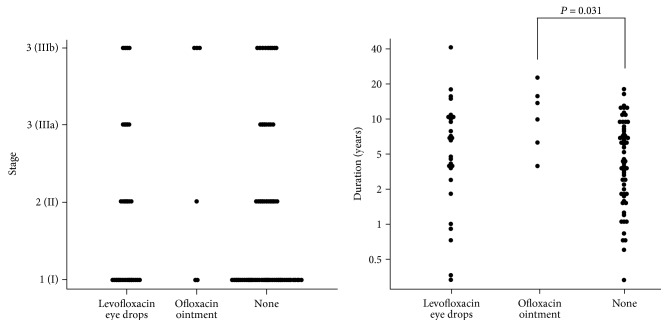
Multiple comparisons of topical antibiotic administration effects on infection onset severity and duration from the last glaucoma surgery to the bleb-related infection onset (Steel test).

The median stage of the steroid-administered eyes was 3 (range, 1–4) versus 1 (range 1–4) for eyes without steroid administration (*P* = 0.012; Table 1). In a multiple comparison analysis, the stage of the 0.1% betamethasone eye drop-administered eyes was 3.5 (range, 2–4) versus 1 (range 1–4) for eyes without steroid administration (*P* = 0.007; Figure 1). The median duration from surgery to infection onset for the steroid-administered eyes was 2.0 years (range, 0.3–9.8 years) versus 5.8 years (range, 0.3–41.4 years) for eyes without steroid administration (*P* = 0.030). In a multiple comparison analysis, the median duration from surgery to infection onset for the 0.1% betamethasone eye drop-administered eyes was 1.8 years (range, 1.4–3.2 years) versus 5.8 years (range, 0.3–41.4 years) for eyes without steroid administration (*P* = 0.049). The median duration from surgery to infection onset for the antibiotic-administered eyes was 6.4 years (range, 0.3–41.4 years) versus 3.9 years (range, 0.3–17.5 years) for eyes without antibiotic administration (*P* = 0.025). In a multiple comparison analysis, the median duration for the 0.3% ofloxacin ointment-administered eyes was 10.5 years (range, 3.6–14.7 years) versus 3.9 years (range, 0.3–17.5 years) for eyes without antibiotic administration (*P* = 0.031; Figure 2). There was no significant effect of sodium hyaluronate use on infection severity and duration from the surgery to infection onset.

### 3.3. Relationships between Topically Administered Steroids, Antibiotics, and Sodium Hyaluronate on IOP, LogMAR VA, Bleb Morphology, Bleb Vascularity, and History of Bleb Leakage Prior to Infection

There was no significant effect of steroid, antibiotic, or sodium hyaluronate use on IOP, and logMAR VA tended to deteriorate irrespective of the use of these agents (Table 3). No patients had inferior located filtering bleb. Steroid and sodium hyaluronate administration was not significantly related to bleb morphology, bleb vascularity, or history of bleb leakage prior to infection. However, the rate of bleb leakage prior to infection in the antibiotic-administered group was significantly higher than that in the group with eyes not administered with antibiotics (*P* = 0.003). There was no significant relationship between the administration of steroids, antibiotics, or sodium hyaluronate on bacterial cultures (Table 4).

### 3.4. Multiple Logistic Regression Analysis to Identify Factors Associated with Infection Severity and Duration from the Surgery to Infection Onset

We categorized the eyes into two groups based on infection severity (stage 1 or stage >1) and the duration from last glaucoma surgery to infection onset based on the median duration (>5.5 years or ≤5.5 years). The associated factors were history of bleb leakage prior to infection, bleb vascularity, and topical administration of steroid, antibiotics, or sodium hyaluronate. The multiple logistic regression analysis revealed that the infection was severe in the steroid-administered eyes (odds ratio, 4.57; 95% confidence interval, 1.17–17.80; *P* = 0.029). Other variables were not included in the logistic regression analysis.

## 4. Discussion

The median infection stage in eyes topically treated with steroids was higher than that in eyes not treated with steroids. In a multiple comparison analysis, the stage of the 0.1% betamethasone eye drop-administered eyes was significantly severe. The median period from surgery to infection onset in the steroid-administered eyes was shorter than that in eyes without steroid administration. In a multiple comparison analysis, the period of the 0.1% betamethasone eye drop-administered eyes was significantly shorter than that in eyes without steroid administration. Conversely, the median period from surgery to infection onset was longer for the antibiotic-administered eyes than that for the eyes without antibiotic administration. Further, the period was significantly longer in the ofloxacin ointment-administered eyes. For 95.2% (40/42) of eyes in which antibiotics were administered, new-generation quinolones were used, and levofloxacin was the most frequently used antibiotic for 66.7% (28/42) of eyes.

All infections occurred 17 weeks or later postoperatively, and all eyes may have become infected near or after completion of surgical wound healing [9, 31]. While the postoperative wound healing process continues, topical steroids are commonly administered for a few months to suppress the strong immune response [9, 18, 19, 32]. Moreover, Starita et al. [6] reported that topical steroid administration for only 20 days in the immediate postoperative period improved the long-term prognosis of filtering surgery, and corticosteroid-treated eyes showed a higher rate of thin cystic bleb formation. Steroids prevent bleb failure by modulating the wound healing process and improve postoperative IOP control [7, 8]. Therefore, steroids may have been administered for a long period to improve IOP for some eyes. Moreover, some eyes needed long-term steroid administration because they had special eye conditions, such as postkeratoplasty and uveitis.

If steroids are administered to eyes with bleb-related infection risk, such as bleb leakage or thin-walled blebs [33–35], the infection risk may be higher due to the immunosuppressive effects of the steroids [19]. In this study, many eyes were avascular and/or had a history of bleb leakage. Therefore, eyes treated with steroids postoperatively for a long period of time may have had severe and earlier onset infections. The immunosuppressive effect of betamethasone is very strong [36], and the retention time in the anterior chamber after administration is long [37, 38]. Therefore, betamethasone may strongly suppress immunity in the anterior chamber for a long period of time, and infection may have easily spread into the anterior chamber and become severe in the betamethasone-administered eyes. Although the exact period of steroid use is unknown, in 84.6% of eyes in which topical steroids were administered, the duration from the most recent visit to infection being detected was longer than two weeks. Ophthalmologists should carefully follow-up patients with vulnerable blebs to avoid the development of bleb-related infections when topical steroids, especially betamethasone, are administered for more than two weeks after the immediate postoperative period. Although most eyes administered topical steroids were also administered antibiotics simultaneously, severe and earlier onset bleb-related infections developed. Therefore, the combined use of antibiotics may be insufficiently effective, or even completely ineffective, in preventing bleb-related infections in patients with avascular or partially avascular blebs.

In this study, the time from surgery to infection onset was longer for the antibiotic-administered eyes. Therefore, topical antibiotic administration may be effective in suppressing earlier onset bleb-related infections. Conversely, Lamping et al. [20] reported that 4/252 eyes had a bleb-related infection after filtering surgery and three of these four eyes had been treated with prophylactic antibiotics; therefore, prophylactic antibiotic use may not have prevented the bleb-related infection. Jampel et al. [21] also reported that in 131 cases of late-onset bleb infection, an intermittent and continuous use of antibiotics after filtering surgery was associated with an increased infection risk. Levofloxacin was only released after 2000 [39, 40], whereas the data analyzed by Lamping et al. and Jampel et al. were collected before 1998; hence, they were unable to use levofloxacin. Levofloxacin possesses superior ocular penetration and strength, and it remains in the anterior chamber for a long period of time [39, 41–43]. Prophylactic administration of new-generation quinolones in eyes with vulnerable blebs may be effective in suppressing earlier onset bleb-related infections. Therefore, further investigation is needed to establish the efficacy of the prophylactic administration of new-generation quinolones in preventing bleb-related infections.

It has been reported that long-term antibiotic usage does not appear to alter the conjunctival flora [22]. In this study, the result of bacterial cultures was similar for eyes that were and were not administered steroids as well as for eyes that were and were not administered antibiotics. However, when antibiotics are administered for a long period of time, ophthalmologists must be aware of the potential appearance of drug-resistant bacteria, and when steroids are administered for a long period of time, they must be aware of opportunistic infections caused by steroid-induced immune suppression [19].

Although no relationship was detected between sodium hyaluronate and the analyzed factors, some relationship may have been detected if the eyes administered sodium hyaluronate eye drops were examined continuously postoperatively.

Inferior location of the filtering bleb is a risk factor of bleb-related infections [15]. Greenfield et al. described that inferior filtering blebs are frequently exposed and poorly covered by the lower eyelid. This may result in a more friable epithelium, secondary to the effects of repeated trauma as the lower eyelid rubs the bleb with each blink [15]. In this study, no patients had inferior located filtering bleb. However, if the blebs are located in the inferior portion, bleb traumatism due to the frequent eye drops or ointments instillations may occur. Moreover, this study only involved patients who had bleb-related infections. To correlate the effects of long-term topical administration of the studied medications and bleb-related infection, a control group should have been included, and the hazard ratio for the use of long-term topical agents on bleb-related infection development should have been presented. Further studies are needed to address this limitation.

## 5. Conclusion

Long-term and topical administration of steroids, especially betamethasone, is related to severe and earlier onset bleb-related infection in eyes with avascular or partially avascular blebs. Certainly, in sufficiently vascular blebs, topical steroids may help with long-term survival of blebs. However, if the blebs are avascular or partially avascular, ophthalmologists should abstain from using long-term topical steroids beyond the immediate postoperative period. If the administration of topical steroids, especially betamethasone, is required for eyes that have vulnerable blebs for a long period of time after glaucoma surgery, alternatives to filtering surgery must be considered. Topical administration of new-generation quinolones, particularly levofloxacin, may be effective for suppressing earlier onset bleb-related infections. Polypharmacy with ofloxacin ointment may increase these effects.

## Figures and Tables

**Table 1:** Relationships among administered agents, infection onset severity, and duration from last glaucoma surgery to bleb-related infection onset.

	*N* (%)	Stage			Duration (y)		
		Median (IQR, min/max)		^∗^ *P*	Median (IQR, min/max)		^∗^ *P*
Steroid							
Yes	13 (12.5)	3.0 (2.0–4.0, 1.0/4.0)	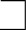	0.012	2.0 (1.1–6.1, 0.3/9.8)	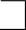	0.030
No	91 (87.5)	1.0 (1.0–3.0, 1.0/4.0)			5.8 (3.0–9.1, 0.3/41.4)		
0.1% BM eye drops	6 (5.8)	3.5 (3.0–4.0, 2.0/4.0)	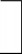	0.013	1.8 (1.4–3.2, 1.1/6.1)	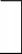	0.036
0.02 and 0.1% FM eye drops	6 (5.8)	1.5 (1.0–2.8, 1.0/4.0)			2.3 (0.5–7.7, 0.3/9.5)		

Antibiotic							
Yes	42 (40.4)	2.0 (1.0–3.0, 1.0/4.0)	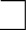	0.362	6.4 (3.5–10.1, 0.3/41.4)	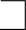	0.025
No	62 (59.6)	1.0 (1.0–3.0, 1.0/4.0)			3.9 (2.0–7.2, 0.3/17.5)		
0.5% LVFX eye drops^a^	28 (26.9)	1.5 (1.0–3.0, 1.0/4.0)	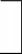	0.375	6.2 (3.2–9.5, 0.3/41.4)	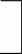	0.049
0.3% OFLX ointment	6 (5.8)	3.0 (1.3-4.0, 1.0/4.0)			10.5 (6.8–12.7, 3.6/14.7)		

Sodium hyaluronate							
Yes	16 (15.4)	2.0 (1.0–3.0, 1.0/4.0)	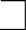	0.649	6.1 (4.1–9.0, 0.9/12.0)	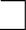	0.351
No	88 (85.6)	1.0 (1.0–3.0, 1.0/4.0)			4.7 (2.2–9.0, 0.3/41.4)		

IQR: interquartile range; BM: betamethasone; FM: fluorometholone; LVFX: levofloxacin; OFLX: ofloxacin. ^∗^*P* value for comparing two groups (Mann–Whitney *U* test) and three groups (Kruskal–Wallis test). ^a^One eye administered with other antibiotics simultaneously are excluded.

**Table 2:** Antibiotics administered, except levofloxacin eye drops alone.

	Other antibiotic eye drops	OFLX ointment	New quinolone
0.5% LVFX eye drops and combination ointment of 0.35% fradiomycin sulfate and 0.1% methylprednisolone	1	—	Yes
0.3% OFLX eye drops	1	—	Yes
0.3% OFLX eye drops and 0.5% cefmenoxime hydrochloride eye drops	1	—	Yes
0.3% Norfloxacin and 0.5% cefmenoxime hydrochloride eye drops	1	—	Yes
0.3% Gatifloxacin eye drops	2	—	Yes
0.5% Cefmenoxime hydrochloride and 1.0% sulbenicillin sodium eye drops	1	—	
0.25% Chloramphenicol and 0.8% colistin sodium methanesulfonate eye drops	1	—	
0.3% OFLX ointment	—	2	Yes
0.3% OFLX ointment and 0.3% OFLX eye drops	—	1	Yes
0.3% OFLX ointment and 0.5% LVFX eye drops	—	2	Yes
0.3% OFLX ointment and combination eye drops of 0.35% fradiomycin sulfate and 0.1% betamethasone	—	1	Yes

OFLX: ofloxacin; LVFX: levofloxacin.

**Table 3:** Characteristics of the eyes administered steroids, antibiotics, or sodium hyaluronate.

	Steroid				Antibiotic				Sodium hyaluronate			
	Yes	*P*1	No		Yes	*P*1	No		Yes	*P*1	No	
	Median (IQR)		Median (IQR)	*P*1	Median (IQR)		Median (IQR)	*P*1	Median (IQR)		Median (IQR)	*P*1
IOP(mmHg)												
Preinfection	17 (13–19)	0.844	10 (8–12)	0.065	11 (8–14)	0.182	9 (7–13)	0.191	11 (7–14)	1.000	10 (8–13)	0.562
Postinfection	19 (15–20)		11 (8–15)		13 (10–18)		11 (8–15)		13 (10–16)		12 (8–16)	

LogMAR VA												
Preinfection	0.8 (0.4–1.2)	0.058	0.2 (0.0-1.0)	0.055	0.5 (0.0-1.5)	0.029	0.1 (0.0-1.0)	0.001	0.5 (0.1–1.8)	0.281	0.2 (0.0-1.0)	0.001
Postinfection	1.7 (1.1-2.9)		0.7 (0.0–2.7)		1.3 (0.1-2.3)		0.4 (0.0–2.7)		1.3 (0.3–2.7)		0.7 (0.0–2.7)	
	*N* (%)		*N* (%)	*P*2	*N* (%)		*N* (%)	*P*2	*N* (%)		*N* (%)	*P*2

Bleb morphology												
Total 100 (100%)	13 (13.0)		87 (87.0)		39 (39.0)		61 (61.0)		16 (16.0)		84 (84.0)	
Cystic	5 (5.0)		42 (42.0)	0.681	19 (19.0)		28 (28.0)	0.506	8 (8.0)		39 (39.0)	0.849
Diffuse	8 (8.0)		40 (40.0)		20 (20.0)		28 (28.0)		7 (7.0)		41 (41.0)	
Encapsulated	0 (0.0)		4 (4.0)		0 (0.0)		4 (4.0)		1 (1.0)		3 (3.0)	
Flat	0 (0.0)		1 (1.0)		0 (0.0)		1 (1.0)		0 (0.0)		1 (1.0)	

Bleb vascularity												
Total 99 (100%)	13 (13.1)		86 (86.9)		38 (38.4)		61 (61.6)		16 (16.2)		83 (83.8)	
Avascular	9 (9.1)		68 (68.7)	0.469	33 (33.3)		44 (44.4)	0.081	12 (12.1)		65 (65.7)	0.818
Partial avascular	4 (4.0)		16 (16.2)		5 (5.1)		15 (15.2)		4 (4.0)		16 (16.2)	
Vascular	0 (0.0)		2 (2.0)		0 (0.0)		2 (2.0)		0 (0.0)		2 (2.0)	

Bleb leakage												
Total 101 (100%)	13 (12.9)		88 (87.1)		41 (40.6)		60 (59.4)		16 (15.8)		85 (84.2)	
Leak (+)	4 (4.0)		37 (36.6)	0.445	24 (23.8)		17 (16.8)	0.003	8 (7.9)		33 (32.7)	0.406
Leak (−)	9 (8.9)		51 (50.5)		17 (16.8)		43 (42.6)		8 (7.9)		52 (51.5)	

IOP: intraocular pressure; VA: visual acuity. *P*1: *P* value for IOP and LogMAR distribution (preinfection versus 12 months postinfection by Wilcoxon signed-rank test). *P*2: *P* value for bleb morphology, bleb vascularity, and bleb leakage variables (Mann–Whitney *U* tests). Avascular: avascular area of the blebs is ≥50%; Partial avascular: avascular area of the blebs is <50% but the blebs are not vascular; Vascular: blebs have no avascular area. Leak (+): number of patients with history of bleb leak prior to infection; Leak (−): number of patients without bleb leak prior to infection.

**Table 4:** Relationship between results of bacterial cultures and administered agents.

	Steroid		Antibiotic		Sodium hyaluronate		Total
	Yes	No	Yes	No	Yes	No	
Culture performed [*N* (%)]	12/13 (92.3)	82/91 (90.1)	38/42 (90.5)	56/62 (90.3)	14/16 (87.5)	80/88 (90.9)	94/104 (90.4)
Culture positive [*N* (%)]	5/12 (41.7)	42/82 (51.2)	19/38 (50.0)	28/56 (50.0)	6/14 (42.9)	41/80 (51.3)	47/94 (50.0)
Strains isolated	5	43	19	29	6	42	48
*S. aureus* (including MRSA)	2	6	4	4	2	6	8
CNS spp. (including MRSE)	0	7	3	4	0	7	7
*Streptococcus* spp.	2	16	7	11	2	16	18
*Corynebacterium* spp.	0	5	0	5	2	3	5
*Enterococcus* spp.	0	3	0	3	0	3	3
*H. influenzae*	0	2	1	1	0	2	2
*Pseudomonas aeruginosa*	0	0	0	0	0	0	0
*Micrococcus luteus*	0	1	1	0	0	1	1
Gram-positive bacillus (Unidentified)	0	1	0	1	0	1	1
Gram-negative bacillus (Unidentified)	1	0	1	0	0	1	1
*Gemella haemolysans*	0	1	1	0	0	1	1
Anaerobic bacteria (Unidentified)	0	1	1	0	0	1	1

CNS: coagulase-negative staphylococcus; MRSA: methicillin-resistant *S. aureus*; MRSE: methicillin-resistant *S. epidermidis.*
